# Cell-mediated and serology-based tests for *Mycobacterium ulcerans* disease: A systematic review and meta-analysis

**DOI:** 10.1371/journal.pntd.0008172

**Published:** 2020-04-06

**Authors:** Michael S. Avumegah, Nilakshi T. Waidyatillake, Wojtek P. Michalski, Daniel P. O’Brien, Tiffanie M. Nelson, Eugene Athan

**Affiliations:** 1 The University of Queensland, School of Chemistry and Molecular Bioscience, Brisbane, Australia; 2 Deakin University, School of Medicine, Geelong Australia; 3 Geelong Centre for Emerging Infectious Diseases (GCEID), Geelong, Australia; 4 Department of Infectious Diseases, Barwon Health, Geelong, Australia; 5 Allergy and Lung Health Unit, Melbourne School of Population and Global Health, The University of Melbourne, Melbourne, Australia; 6 Commonwealth Scientific and Industrial Research Organisation, Australian Animal Health Laboratory (CSIRO AAHL), Geelong, Australia; 7 Department of Medicine and Infectious Diseases, Royal Melbourne Hospital, The University of Melbourne, Melbourne, Australia; Hospital Infantil de Mexico Federico Gomez, UNITED STATES

## Abstract

Buruli ulcer (BU) is a subcutaneous necrotic infection of the skin caused by *Mycobacterium ulcerans*. It is the third most common human mycobacterial disease after tuberculosis (TB) and leprosy. The available methods for detection of the bacilli in lesions are microscopic detection, isolation and cultivation of the bacterium, histopathology, and polymerase chain reaction (PCR). These methods, although approved by the World Health Organization (WHO), have infrastructural and resource challenges in medical centres and cell-mediated immunity (CMI) and/or serology-based tests have been suggested as easier and more appropriate for accurate assessment of the disease, especially in remote or underdeveloped areas. This study systematically reviewed and conducted a meta-analysis for all research aimed at developing cell-mediated immunity (CMI) and/or serology-based tests for *M*. *ulcerans* disease. Information for this review was searched through PubMed and Web of Science databases and identified up to June 2019. References from relevant articles and reports from the WHO Annual Meeting of the Global Buruli Ulcer Initiative were also used. Twelve studies beginning in 1952, that attempted to develop CMI and/or serology-based tests for the disease were identified. These studies addressed issues of specificity and sensitivity in context of antigen composition as well as study heterogeneity and bias. The two main types of antigenic preparations considered were pathogen-derived and recombinant protein preparations. There was slight difference in test performance when *M*. *ulcerans* recombinant proteins [positivity: 67.5%; 32.5%] or pathogen-derived [positivity: 76.0%; 24.0%] preparations were used as test antigens among BU patients. However, pathogen-derived preparations were better at differentiating between patients and control groups [odds ratio (OR) of 27.92, 95%CI: 5.05–154.28]. This was followed by tests with the recombinant proteins [OR = 1.23, 95%CI: 0.27–5.62]. Overall, study heterogeneity index, I^2^ was 92.4% (p = 0.000). It is apparent from this review that standardisation is needed in any future CMI and/or serology-based tests used for *M*. *ulcerans* disease.

## Author summary

Buruli ulcer (BU) is a debilitating skin infection caused by *M*. *ulcerans*. It is the third most common mycobacterial disease after tuberculosis and leprosy. BU is mainly restricted to the tropical and subtropical countries of the world, though temperate regions report sporadic cases. Polymerase chain reaction targeting IS*2404* is the gold standard for *M*. *ulcerans* disease diagnosis and other methods such as histopathology, acid fast staining and microscopy are used for validity checks. The currently approved diagnostic tools lack sensitivity and specificity and there are many resource challenges in underdeveloped regions. Isolation and culture of the bacillus from tissue biopsies is the only method that detects viable cells. However, the long incubation period of the pathogen makes it not ideal and rapid enough for point-of-care diagnosis. Cell-mediated immunity and serology-based methods have been suggested as appropriate tools for accurate and rapid testing for the disease within “at-risk-communities”. This study systematically reviewed and conducted a meta-analysis on all research aimed at developing cell-mediated immunity and/or serology-based tests for *M*. *ulcerans* disease.

## Introduction

Buruli ulcer (BU) is a disease caused by the bacteria *Mycobacterium ulcerans* and has been reported in the tropics and sub-tropics of over 33 countries [1], with very few cases reported in temperate areas [2, 3]. Disease progression is marked by destruction of the subcutaneous skin layer, which sometimes damages nerves and blood vessels [4, 5]. The destructive nature of the disease has been attributed to mycolactone, a macrolide toxin produced by the bacillus which causes apoptosis of cells [6, 7]. Although a recent report indicates a 64% reduction in BU cases globally in the last 9 years [1], the incidence in Australia has increased 248% within the same time period [1]. A steady decline has been observed in endemic areas of West Africa (Ghana, Benin and Cote d’Ivoire) except in Nigeria where cases appear to be on the rise [1, 8].

World Health Organization (WHO)-approved laboratory methods for detecting bacilli in lesions include: microscopic detection of acid-fast bacteria (AFB), isolation and cultivation of *M*. *ulcerans*, histopathology and polymerase chain reaction (PCR) for *M*. *ulcerans* insertion sequence IS2404 [9]. Among these methods, IS2404 PCR is considered the gold standard and is routinely used in laboratories for BU confirmation [9]. Microscopy is also used but lacks sensitivity and specificity, and histopathology is labour intensive (11). Nevertheless, both have been used for validity checks. The only method that detects viable bacilli is the isolation and cultivation of *M*. *ulcerans* on Löwenstein–Jensen (LJ) and Middlebrook media at 29–33°C [9]. However, this requires a duration of 9 to 12 weeks or even longer [9, 10]. Due to the complexity of the disease and difficulty with its diagnosis, WHO recommends two laboratory tests for BU confirmation in endemic settings [9].

A previous systematic review by Sakyi *et al*., 2016 [11] provided an in-depth review on various clinical and laboratory methods used to screen for *M*. *ulcerans* infection or to diagnose BU. Techniques discussed included microscopy, culture of the bacilli, histopathology, targeted PCR (including dry reagent based, real-time PCR and nested PCR) and loop mediated isothermal amplification (LAMP assay), thin layer chromatography for mycolactone detection, and serology [11]. All these tests require trained staff with access to well-equipped laboratory facilities [9].

Furthermore, there are difficulties in the accurate diagnosis of BU in endemic localities with the current diagnostic tools [12], resulting in diagnosis delay that inevitably leads to severe forms of BU and long hospitalisation [12, 13]. There is a call to develop an easy to use, reliable and rapid test for BU diagnosis and surveillance in “high-risk communities” to aid timely and effective treatment [12]. Methods based on CMI and serology are thought to be reliable in principle for effective diagnosis. However, previous studies have found that in both approaches cross-reactivity with other mycobacterial infections was difficult to overcome [14, 15]. In addition, immunosupression caused by mycolactone appears to inhibit the full expression of CMI and humoral immune responses in infected individuals [16, 17], that could have been important as biomarkers for BU screening. Cytokine response techniques (CMI-based diagnosis) are still used to screen individuals suspected of infection with *M*. *tuberculosis*, the causative agent of the highly fatal disease, tuberculosis (TB) [18]. Although there has been cross-reactivity with the TB bacilli in *M*. *tuberculosis* infected patients, CMI and serology-based methods for BU screening have been explored by some researchers [15, 19].

Unlike TB, where latent form has been described [20, 21], latent form of *M*. *ulcerans* disease has not been defined. *M*. *ulcerans* has not been isolated from an individual without clinical symptoms. It appears that subclinical BU infection and active BU disease cannot be differentiated by serological or immunological testing at present.

The aim of this study is not to justify the use of CMI or serology-based methods over currently available ones. Our objective is to systematically review and conduct a meta-analysis on original research studies that aimed to develop CMI and/or serology-based tests for *M*. *ulcerans* disease, from various countries, and with different antigen preparations. We provide meta-analysis on the diagnostic potential of different preparations. We also discuss the prospects and challenges of these methods and provide perspectives on future research in this area.

## Methodology

This review was conducted according to the Preferred Items for Systematic Reviews and Meta-Analysis (PRISMA).

### Data sources and search strategy

The following online reference databases were searched for relevant articles: PubMed (1930 to June 2019), Web of Science / ISI Web of Knowledge (1930 to June 2019) and the Buruli ulcer disease database maintained by WHO in Geneva, Switzerland [22]. The search terms used were: “Buruli ulcer and/or *Mycobacterium ulcerans*” in combination with the following terms: “cell-mediated screening test”, “serological screening test” and “diagnostic tests”. The details of conducted searches are included in the supplement (S1 Text).

### Inclusion and exclusion criteria

We included original human and animal studies with full text (or abstract if full text was not available). There was no language restriction set for the searches, however all relevant studies were in English.

### Data extraction and analysis

Study rationale, study design, year of study, *M*. *ulcerans* strain, participant description, country of the study, as well as sensitivity and specificity results were extracted from selected articles to a standard table. Data collation followed the guidelines for review structure in the PRISMA checklist [23] (S1 PRISMA Checklist). The extracted data was cross-checked with a second reviewer (TMN) and any discrepancies were resolved by a senior author (NTW).

### Quality of the included studies

The Newcastle-Ottawa Scale (NOS) was used to assess the quality of the included studies (S1 Data) [24]. NOS is used to assess the quality of non-randomised studies, and scored as follows: Good quality score: 3 or 4 stars in selection domain and 1 or 2 stars in comparability domain and 2 or 3 stars in outcome/exposure domain. Fair quality score: 2 stars in selection domain and 1 or 2 stars in comparability domain and 2 or 3 stars in outcome/exposure domain. Poor quality score: 0 or 1 star in selection domain or 0 star in comparability domain or 0 or 1 star in outcome/exposure domain [24].

### Statistical and meta-analysis

Studies that provided sufficient information, effect size (diagnostic odd ratio) with standard error (SE) were calculated and assigned weight at 95% confidence interval (CI). STATA statistical tool was then used to conduct a random-effects model meta-analysis to assess study heterogeneity and bias. The meta-analysis results were graphically displayed as forest and funnel plots (p< 0.05).

## Results

**Fig 1:**
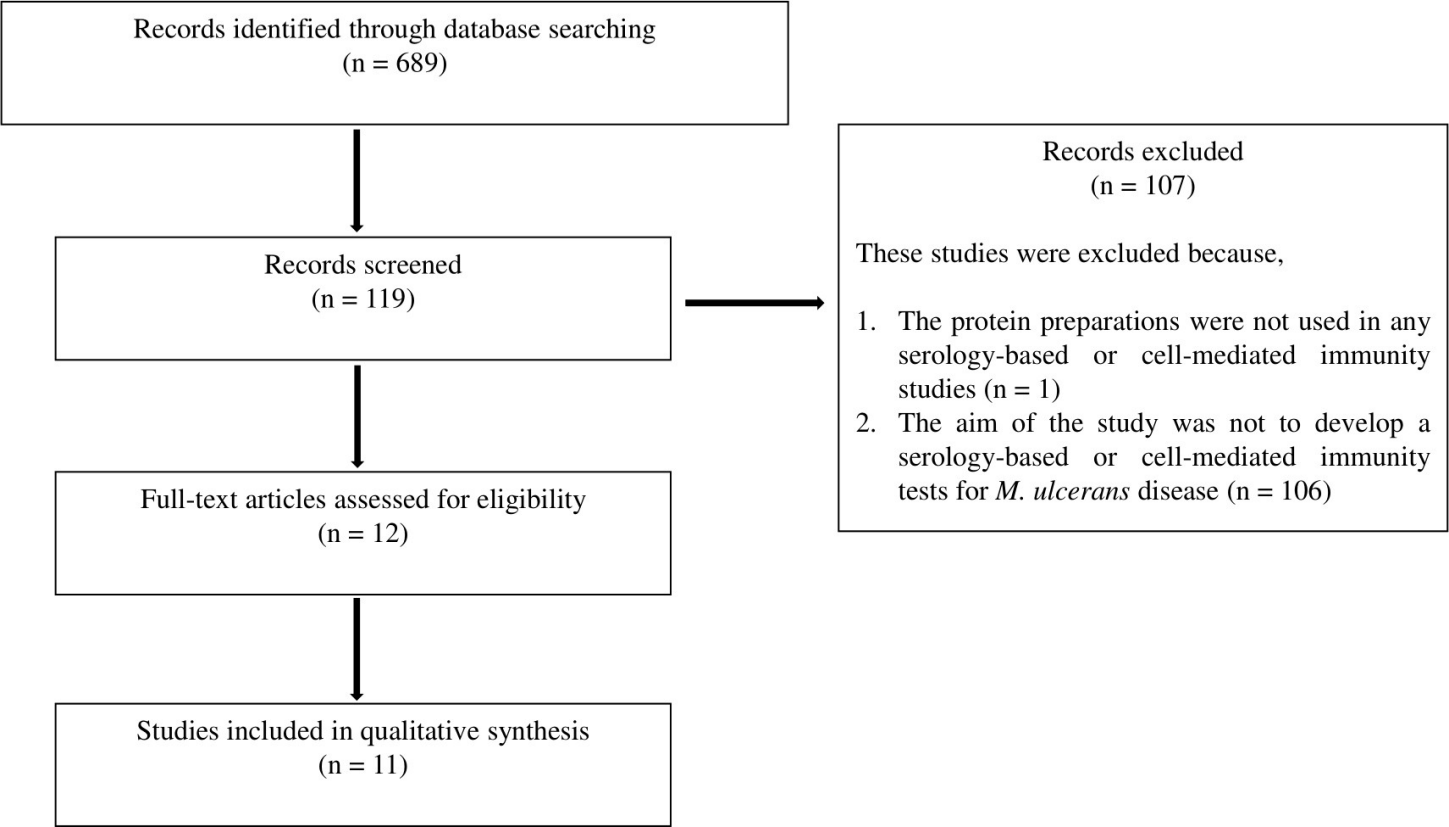
Schematic selection of review articles.

Out of 689 records identified through database searching (n = 689), a total of 119 publications were selected for full text review. After completion of full text review, 107 records were excluded because no defined protein preparation was used in those studies, or the study did not aim to develop CMI or serology-based tests for the disease. Twelve original research studies met the inclusion criteria (Fig 1 and Table 1), with the earliest from 1952 [25] and the most recent from a WHO conference abstract in 2019 presented by Avumegah [8], which was also part of a PhD thesis [26]. Yeboah-Manu *et al*. [15] study in 2012 was reported in our systematic review but we could not include it in the qualitative analyses due to differences in timelines of participant group recruitments. A summary of these 12 studies are presented in Table 1.

**Table 1:** Summary of materials and methods used in reviewed studies.

Study/year	Country	*M*. *ulcerans* strain used	Culture medium	Protein preparation and methodology	Immunodetection analysis	Unique biomarkers	Samples used
**Fenner *et al*. 1952 [25]**	Australia	*SF*, *RS and RT*	LJ, Dubos broth	Heat-killed *M*. *ulcerans* were used to challenge rabbits to produce anti-sera for complement fixation test. Guinea pigs were the source of complement for the test.	*M*. *ulcerans* lysate was used in complement-fixation test.	*M*. *ulcerans* lysate	Rabbit serum, guinea pig and sheep erythrocytes
**Stanford *et al*. 1975 [27]**	Uganda and Zaire	Uganda: nos. 297 and 298 Zaire: no.408	LJ	Mechanical disruption of *M*. *ulcerans* (ultrasonic disintegration) was used for protein preparation. The mixture was centrifuged and the supernatant collected for skin test.	*M*. *ulcerans* lysate was used for a skin test.	Burulin	Human skin test
**Robert *et al*. 1997 [28]**	Australia	Not stated	LJ, Dubos broth	Mechanical disruption of *M*. *ulcerans* (constant aggitation) was used for protein preparation. The mixture was centrifuged and the crude culture filtrates (CCF) was havested for the cytotoxicity assay.	CCF was used for *in vitro* and *in vivo* cytotoxic assays in animal model (rabbits and mice).	Mycolactone	Mice footpad and human serum
**Dobos *et al*. 2000 [29]**	Cote d’Ivoire	Lyophilised *M*.*ulcerans* S-WT strain (from the Centres for Disease Control and Prevention culture collection; Atlanta).	MB 7H9 (OADC). 7H9TG broth	No mechanical disruption of Bacillus. *M*. *ulcerans* was cultured in protein and serum free media. Mid-log phase *M*. *ulcerans* culture was centrifuged and supernatant [*M*. *ulcerans* culture filtrate (MUCF) ] was concentrated at 4°C under Nitrogen.	MUCF and PPD samples were used in human skin test and western blotting.	70 kDa, 38/36 kDa, 5 kDa protein	Human skin test and serum
**Okenu *et al*. 2004 [30]**	Ghana	Study stated *M*. *ulcerans* culture filtrate (MUCF) preparation followed Dobos *et al*. protocol [29].	Study stated *M*. *ulcerans* culture filtrate (MUCF) preparation followed *Dobos et al*. protocol [29].	*M*. *ulcerans* culture filtrate (MUCF) preparation followed Dobos *et al*. [29]. MUCF was resolved by sodium dodecyl sulfate polyacrylamide gel electrophoresis by (SDS-PAGE).	MUCF was used in western blot.	MUCF	Human serum (IgM)
**Diaz *et al*. 2006 [19]**	Ghana	The *M*.*ulcerans* strains used: Democratic Republic of Congo (5151), Angola (960657), Ghana (97–483), Australia (Institute of Tropical Medicine (ITM) 5147, ITM 9540, ITM 9550, and 94–1324), Mexico (ITM 5114), Malaysia (941328), French Guiana (ITM 7922), and Japan (ITM 8756).	Modified LJ and MB 7H10	Mechanical disruption (bead beating) of *M*. *ulcerans* and heat inactivation were employed for protein protein preparation. *M*. *ulcerans* lysate was centrifuged and supernatant was haversted for analyses. Recombinant technology was then used to purify *M*. *ulcerans* immunodominat protein.	Immunodominant proteins were recognised in the *M*. *ucerans* lysates by western blot and enzyme-linked immunosorbent assay (ELISA) on human and animal sera.	18kDa	Human serum
**Pidot *et al*. 2010 [14]**	Benin	*M*. *ulcerans* Agy99 (GenBank accession NC_008611) genome was compared with other mycobacteria species of GenBank accession number; NC_010397, NC_002944, NC_008769, NC_009338, NZ_ABIN00000000, NC_ACBV00000000, NC_002677, NC_010612, NC_008596, NC_008705, NC_009077, NC_008146, NZ_AAKR00000000, NC_002755, NC_009565, NC_000962, NC_009525, NZ_AASN00000000, NC_008595, NC_008726.	Not stated/Not applicable	Using bioinformatics and BLAST, the genomes of *M*. *ulcerans* were compared with other mycobacteria species and *M*. *ulcerans* specific gene constructs were generated. Recombinant technology was then used to express and purifiy these constructs.	Immunogenicity of *M*. *ulcerans* proteins were verified using ELISA.	MUP045, MUP057, MUL_0513, Hsp65, AT- propionate, and KR A, 18 kDa.	Human serum
**Yeboah-Manu *et al*. 2012 [15]**	Ghana	Not stated/Not applicable	Not stated/Not applicable	The samples (18 kDa shsp) used in Diaz *et al*. [19] studies was carried forward into this study.	Antibodies present in sera were analysed using western blot and ELISA.	Biomarker screened for was the *M*.*ulcerans* Anti-18 kDa protein.	Human serum
**Röltgen *et al*. 2014 [31]**	Ghana and Cameroon	Not stated/Not applicable	Not stated/Not applicable	The samples (18 kDa shsp) used in Diaz *et al*. [19] studies was carried forward into this study.	The methods used for sera analyses were western blot and ELSIA.	Biomarker screened for was the *M*. *ulcerans* Anti-18 kDa protein.	Human serum
**Nausch *et al*. 2017 [32]**	Ghana	*M*. *ulcerans* 1 isolate of African origin as described in Philips *et al*. [33].	LJ slopes and Sauton's medium as described in Philips *et al*. [33]	Mechanical disruption of *M*. *ulcerans* as described in Philips *et al*. [33] was used for protein preparation. *M*. *ulcerans* lystate was used for blood stimulation.	Flow cytometry was used to analyse *M*. *ulcerans* stimulated blood samples.	CD4+ T cells (TNFα, IFNγ and CD40L)	Human blood
**Loglo *et al*. 2018 [34]**	Ghana	*M*. *ulcerans* recombinant DNA were constructed at the University of Melbourne, Australia. Ag85A (MUL 4987) was supplied by Dr. G. Pluschke (Swiss Tropical and Public Health Institute, Basel, Switzerland).	Not stated	Recombinant technology was used to express and purifiy 11 *M*. *ulcerans* gene constructs. Purified proteins were used to stimulate whole blood samples from BU patients, endemic and non-endemic.	Interferon-gamma (IFN-γ) and Interleukin 5 (IL-5) ELISA.	PMA, recombinant ACP2, ACP3,Atac1,Atac2,ATp,ER, KR A, KR B, KS C, Ksalt, DH, Ag85Aulc	Human serum and whole blood
**Avumegah 2018 [26]**	Australia	*M*. *ulceran* strian from Victoria	BBA, MB 7H9G and MB 7H9TG broth.	Mechanical disruption (beads beating), heat and non-heat treatments, filtration and acid precipitation of secreted proteins of *M*. *ulcerans* cultures was employed for lystates preparation. Recombinant technology was also used to express and purifiy 4 *M*. *ulcerans* gene constructs previously described by Pidot *et al*. [14].	The lysates and recombinants proteins used western blot and ELSIA to screen for *M*. *ulcerans* specific antibody responses in BU patients, endemic, and non-endemic controls.	*M*. *ulceran* protein preparations in 7H9TG *M*. *ulceran* protein preparation in 7H9G. HSP_65, MUL_2232, MUP_057 and AT-propionate.	Human serum

Table 1 has been adapted from PhD thesis available online [26].

**List of abbreviations not in text**: Acyl carrier protein 2& 3(ACP2, ACP3); Acyltransferase with acetate specificity type 1 &2 (ATac1, ATac2); Acyltransferase with propionate specificity (ATp); Enoylreductase (ER); Ketoreductase type A and B (KRA, KR B); Ketosynthase type C (KS C); Ketosynthase domain (Ksalt), dehydratase (DH), and Phorbol 1-myristate 1-acetate (PMA) [34]. Cluster of differentiation 4 (CD4); CD 40 Ligand (CD40L); Tumor necrosis factor alpha (TNFα) [32].

Nine out of the 12 CMI and/or serological studies identified were conducted in Africa, specifically Benin [14], Cameroon [31], Cote d’Ivoire [29], Zaire (now Democratic Republic of Congo, DRC) [27], Ghana [15, 19, 30–32, 34] and Uganda [27]. The remaining three studies, were conducted in Australia [8, 26, 28].

It is apparent from Table 1 that different *M*. *ulcerans* strains were used for protein preparations across all compared studies [15, 19, 25–32, 34, 35]. The culture media used for *M*. *ulcerans* cultivation were diverse and included: LJ media [25, 27, 28], Dubos broth [25, 28], Middlebrook (MB) 7H9 (Oleic Albumin Dextrose Catalase) [29], MB 7H9 liquid medium with tryptose and glucose [8, 26, 29], MB 7H9 liquid medium with glucose [8, 26], Modified LJ [19], MB 7H10 [19] and Brown and Buckle Agar (BBA) [8, 26].

All 12 studies started with the production of diverse antigens of the infective organism *M*. *ulcerans*. The antigens used for the detection assays were either pathogen-derived proteins (PDP) [26–30, 32], recombinant proteins (RCP) [8, 14, 15, 19, 26, 31] or both [26]. The pathogen-derived preparations were produced by heat-inactivation of *M*. *ulcerans* cells [8, 25, 26] or by mechanical disruption (sonication, agitation or homogenization) [26–28, 32, 36]. The objective of the Fenner *et al*. study was to identify the antigenic structural difference between *M*. *ulcerans* and a group of mycobacterial species (*M*. *tuberculosis*, *M*. *muris*, *M*. *avium*, *M*. *ranae* and *M*. *phlei*) by complement fixation assay [25]. Murine models were used for Fenner’s assay [25] as did the mycolactone cytotoxic assay by Roberts *et al*. [28]. However, in the studies by Stanford *et al*. [27] and Dobos *et al*. [29], a pathogen-derived preparation called Burulin [27] was used as skin test for delayed-type hypersensitivity (DTH) response. This was measured by induration on the skin. Other immunodetection methods used were ELISA [8, 14, 19, 26, 30, 32, 34], Western blot for *M*. *ulcerans* antibodies and cytokine specific screening [8, 19, 26, 29, 31].

All the studies that used BU patient samples for their assay also included healthy controls [8, 14, 15, 19, 26, 27, 29, 31, 32]. It was only Stanford *et al*. [27], Dobos *et al*. [29] and Okenu *et al*. [30] that sought to address the cross-reactivity challenge by including patients other than BU with TB and leprosy, in their assays.

Dobos *et al*. [29] was the first study to report 5 kDa, 36/38 kDa and 70 kDa *M*. *ulcerans* proteins detected by BU patients’ sera in Western blots [29]. Six years later, Diaz *et al*. also reported the *M*. *ulcerans* immuno-dominant 18 kDa small heat shock protein (18 kDa shsp) [19], which has since been used in several sero-epidemiology studies including those of Pidot *et al*. [14], Yeboah-Manu *et al*. [15], Röltgen *et al*. [31] and Avumegah [8, 26]. Four years after the identification of the 18 kDa shsp, Pidot *et al*. also identified 45 *M*. *ulcerans*-specific antigens through a genomic study of 20 mycobacterial species compared with *M*. *ulcerans* (Agy99 strain), of which 33, 18 kDa shsp inclusive were successfully purified and used in a serological study. Included in this study were the *M*. *ulcerans* specific proteins (18 kDa shsp, MUP045, MUP057, MUL_0513, ATP and KRA) and the mycobacteria ubiquitous chaperon protein (Hsp65). Surprisingly Hsp65 [8, 14, 26, 37], was better at discriminating BU endemic groups from healthy controls. Out of the 12 studies selected for review, five utilised the 18 kDa shsp protein [8, 14, 15, 19, 26, 31]. The CMI study by Nausch *et al*. also reported on the TNFα+CD40L-IFNγ- CD4+ T cell subset for BU diagnosis [32]. In 2018, Loglo *et al*.,used PMA, recombinant ACP2, ACP3, Atac1, Atac2, ATp, ER, KR A, KR B, KS C, Ksalt, DH, Ag85Aulc antigens as stimulant for IFN-γ and IL-5 expression ELISA assays [34].

### Reactivity of CMI and serology-based tests among BU patients and controls

Positive immune responses to *M*. *ulcerans* recombinantly generated, and pathogen-derived antigens results are presented in Table 2. Positive reactivity among the BU patients ranged from 70% to 85% when using pathogen-derived preparations, but the range of reactivity in controls varied greatly from 3 to 37%. The worst performing recombinant protein was ACP3, showing 47% reactivity among BU patients [34]. Also the panel of proteins used in the CMI study by Loglo *et al*. [34], could not differentiate BU patients from healthy controls [34], however, the 18 kDa shsp was by far a better test antigen.

**Table 2:** Comparison of proportion positive for BU patients and controls.

Studies	Protein preparations	BU patients			Controls			Comparison of proportion positive for BU patients versus controls		
		N	Positive	Negative	N	Positive	Negative	OR	95% CI	p-value
**Pathogen-derived preparations (PDP)**										
**Stanford *et al*. (1975) (Uganda and DRC)**	Burulin (low concentration)	15	12 (80%)	3 (20%)	751	25 (3%)	726 (97%)	116.20	33.49–403.12	< 0.001
**Dobos *et al*. (2000), Ghana**	Cultural filtrate (serology)	61	43 (70%)	18 (30%)	27	10 (37%)	17 (63%)	4.06	1.65–10.02	0.005
	Burulin (DTH)^#^	39	28 (72%)	11 (28%)	21	3 (14%)	18 (86%)	15.27	4.02–58.02	< 0.001
**Okenu *et al*. (2004), Ghana**	Culture filtrate (IgM serology)	66	54 (85%)	12 (15%)	66	3 (5%)	63 (95%)	94.50	27.36–326.34	<0.001
**Total**		181	137 (76%)	44 (24%)	865	41 (5%)	824 (95%)			
**Recombinant protein (RCP)**										
**Diaz *et al*. (2006), Ghana**	18 kDa	32	24 (75%)	8 (15%)	24	9 (38%)	15 (62%)	5.00	1.62–15.41	0.007
**Loglo *et al*. 2018, Ghana**	PMA, recombinant ACP2, Atac1,Atac2,ATp,ER, KR A, KR B, KS C, Ksalt, DH,Ag85Aulc	24	> 19 (80%)	< 5 (20%)	41	> 32 (80%)	< 9 (20%)	1.07	0.34–3.34	1.000
	ACP3*	24	11 (47%)	13 (53%)	41	29 (71%)	12 (29%)	0.35	0.12–1.04	0.065
**Total**		80	54 (67.5%)	26 (32.5%)	106	70 (66%)	36 (34%)			

The studies by Fenner *et al*. [25], Pidot *et al*. [14], Roberts *et al*. [28], Röltgen *et al*. [31] and Avumegah [8, 26], reported insufficient results to extract meaningful data on the number of positive and/or negative samples and as such were excluded from Table 2. Pidot *et al*. [14], however, reported an average sensitivity of 69% and a specificity of 88% for their top six antigens [14]. It must also be pointed out that in this study, sensitivity and specificity values were calculated based on ELISA results of “BU endemic” (ELISA absorbance values of both BU patients + healthy endemic controls) against non-endemic controls [14]. This was based on the assumption that healthy endemic controls living in the same area as BU patients could equally be exposed to *M*. *ulcerans* without obvious symptoms of disease [14]. Yeboah- Manu *et al*. [15] shared the same assumption. Avumegah [8, 26], who used the same recombinant proteins (ATP, Hsp65, MUP057 and MUL_2232) described by Pidot *et al*. [14] as test antigens in ELISA and Western blots in an Australian cohort did not find significant difference between BU patients and healthy controls with regards to specificity. The non-heat treated pathogen preparation used as antigen in ELISA was able to discriminate between BU patients from the control group with 90% sensitivity (CI = 55.50–99.75%) and a specificity of 95% (CI = 75.13% - 99.87%) [8, 26]. This antigen, however, was not used on TB or leprosy patients to check for cross-reactivity. On the other hand, serum antibody responses to the heat treated antigenic preparations were highly variable and lacked specificity [8, 26].

### Meta-analysis

**Fig 2:**
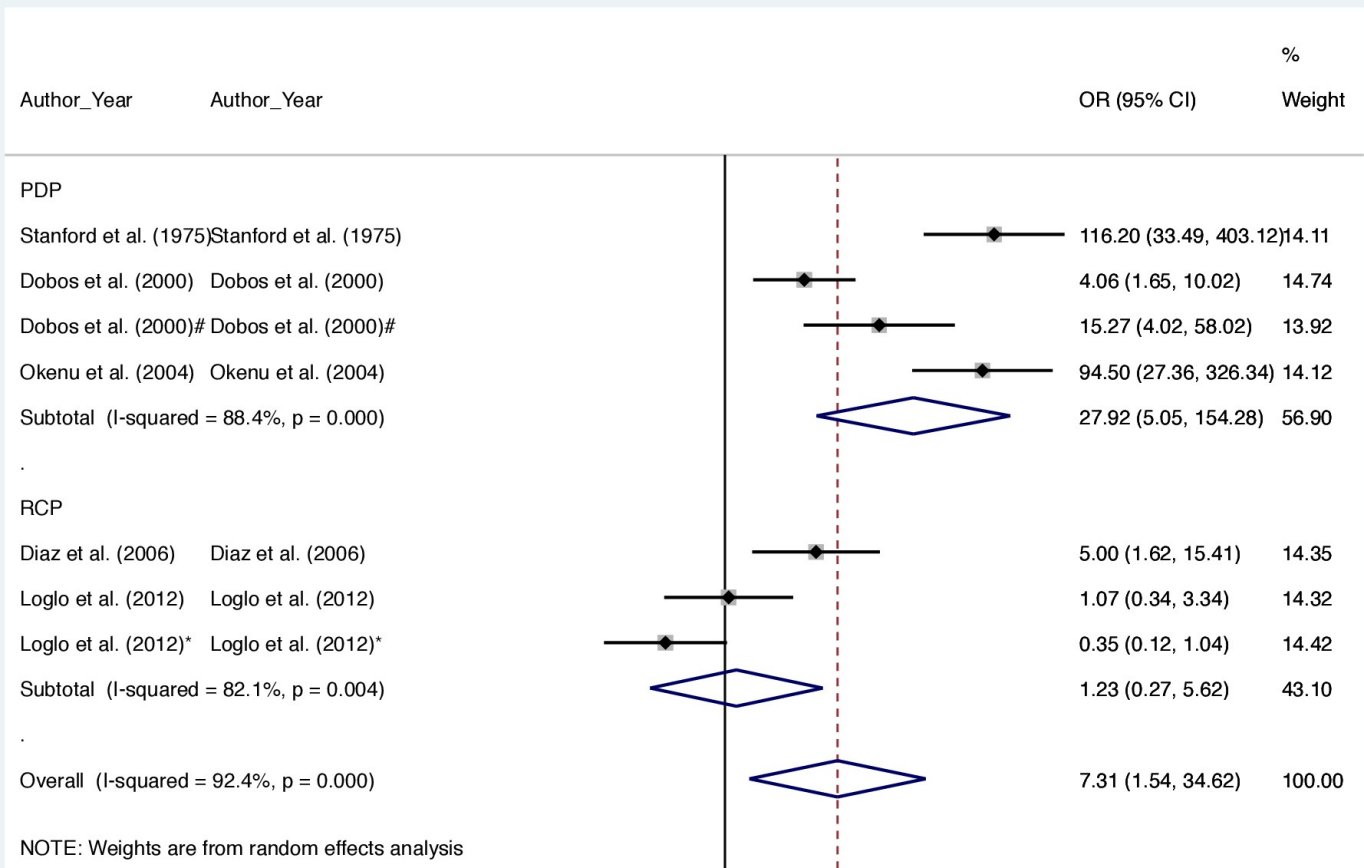
Summary plot of random-effects meta-analysis of 7 studies that have attempted developing a cell-mediated or serology-based test for BU disease. The solid diamond ♦ show the mean odds ratio for each study. ≅The subgroup heterogeneigty index (I^2^) for PDP and RCP studies were > 80%, p = 0.000. The overall I^2^ for entire studies was 92.4%, p = 0.000, and this is shown as ◊. All 7 studies had an average weight > 13.00% (range: 13.92–14.74).

**Fig 3:**
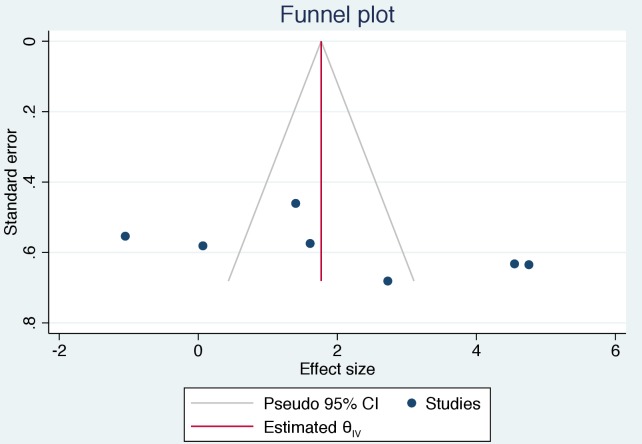
Funnel plot comparing 7 studies that have attempted developing cell-mediated and/or serology-based tests for BU disease. The y-axis is the standard error (SE) measuring precision and the x-axis is the diagnostic odd ratio (DOR). Each of the studies is represented as blue solid circle ●. The solid vertical line measures bias. The grey diagonal dash lines is a pseudo 95% confidence limit of the bias measurment.

The largest odds of BU positive response versus controls was observed in studies where tests were based on pathogen-derived preparations [OR = 27.92, 95%CI: 5.05–154.28], as compared with those based on recombinant antigens [OR = 1.23, 95%CI: 0.27–5.62]. There was significant heterogeneity among the reviewed studies (Fig 2). Studies utilising PDP and RCP as test antigens both reported I^2^ > 80% (range: 82.1% - 88.4%, p = 0.000). The overall heterogeneity among studies was 92.4% (p = 0.000). Funnel plot (Fig 3) with pseudo 95% confidence limits revealed a clear asymmetric relationship between studies.

## Discussion

This systematic review and meta-analysis summarises the results of 12 studies which looked at developing CMI and serology-based assays for *M*. *ulcerans* disease. Our review and meta-analysis show that the experimental design for CMI or serology-based tests for BU varies greatly. In addition, there appears to be a significant heterogeneity (I^2^ = 92.4%) in the test outcomes. Disparity in outcome was not entirely unexpected, which was also confirmed by the asymmetric funnel plot. It is unlikely this was due to publication bias alone. The heterogeneity and bias in the effects of PDP and RCP as test antigens could be due to differences in *M*. *ulcerans* strains used for protein preparations, protein production procedures, study populations and type of samples tested. There is the urgent need for standardisation of study design in BU. It would be appropriate for future studies involving PDP preparation to consider conducting mass spectrometric analysis of protein composition for comparative studies as did Avumegah [8, 26]. It is fair to assume that since pathogen-derived antigenic preparation is a mixture of whole bacterial lysate, it would be nearly impossible to elucidate the most reactive components in cell-mediated or humoral immune response experiments without fractionation or purification. The effects of immuno-dominant and highly expressed proteins could curtail the ultimate effect of critical but low expressed proteins.

The only systematic review that is similar to our study was that by Sakyi *et al*. [11]. There was no discussion on CMI or serology-based assays other than that of Dobos *et al*. [29]. Therefore, we have provided a detailed overview of CMI and serology-based studies aimed at developing detection assays for *M*. *ulcerans* disease.

Immune modulation by mycolactone [16, 38, 39] could be one of the reasons CMI and serology-based methods in BU have not been popular. However, our review has provided an insight and general overview of studies that have shown promising results and that need to be further validated. There appears to be a lack of research continuity between approaches to arrive at meaningful conclusions. For example, Stanford’s skin test study with burulin in Uganda and DRC [27] has not been replicated elsewhere. The same could be said about skin test in Dobos *et al*. study in Côte d’Ivoire with *M*. *ulcerans* cultural filtrates [29]. These two skin tests [27, 29], albeit similar, were not comparable as they used different *M*. *ulcerans* strains and different protein preparation procedures. We are aware of the limitation of the potential “small sample size effect” (only 12 studies were reviewed) in exaggerating bias in our meta-analysis. However, this was beyond our control and further highlights the need for more research efforts in this area.

It is obvious there is no approved standard or study design for conducting BU CMI or humoral immunity studies. Furthermore, there is also no agreed upon test antigen or *M*. *ulcerans* strain to use. We assessed the quality of the individual studies using the Newcastle-Ottawa Scale (S1 Data) [24].

### Way forward and research perspectives

There is a clear need for *M*. *ulcerans* strain selection from which protein preparations would be made for comparable and consistent CMI and humoral experiments. Screening tests based on pathogen-derived preparation and recombinant protein expression have also showed promise, but experimental optimisation and standardisation are key. The fact that pathogen-derived preparations were better at discriminating between BU patients and controls is interesting. This could mean that among the *M*. *ulcerans* whole cell lysates, some unique pathogen-specific proteins are expressed and these can serve as lead candidates antigens in CMI or serology-based assays. Recombinant proteins identified could be used in combination with pathogen-derived preparations for screening purposes. A recent publication on *M*. *tuberculosis* point-of-care screening for wild animals has successfully used a combination of *M*. *tuberculosis* specific antigens as well as precipitated protein derivates (PPD) [40].

It is important to keep in mind that there will be differences in expressed pathogen antigens between *M*. *ulcerans* within the host and during in vitro experiments. Once infective within a host, *M*. *ulcerans* may potentially up-regulate particular proteins of immunogenic importance that may never be expressed in vitro (culture medium) due to differences in interactions and growth environment. Therefore, it may also be necessary to culture *M*. *ulcerans* under different growth conditions and assess the composition of cell-derived protein preparations by mass spectrometric analyses for comparative studies. This proposition is important as *M*. *ulcerans* 18 kDa shsp and other heat shock proteins/stress biomarkers are up- or down-regulated during certain adverse conditions to protect cells [41, 42]. There is a need for a comprehensive genomic and proteomic evaluation among different *M*. *ulcerans* strains and other mycobacterial species to further assess this phenomenon.

In addition, normalisation of total immunoglobulin in sera from study participants could provide an opportunity to compare immune responses across study groups. It is not unusual to develop hypergammaglobulinemia (a condition of elevated immunoglobulin) during active infection with pathogenic bacteria [43], which could result in false positives. Moreover, the establishment of ELISA absorbance ranges or cut-offs for BU or *M*. *ulcerans* sero-positive indviduals would be an ideal goal of future studies.

PCR has proven to be one of the most effective diagnostic methods for clinical disease in health settings where it is affordable, however other methodologies became recently available. Two alternative methods based on specific microRNA (miRNA) and mycolactone appear to warrant further investigation. MiRNA are short non-coding RNA sequences usually between 2–22 nucleotides. They are synthesised from longer nucleotides from animals, plants and virus genomes as a result of post-transcriptional repression of gene expression [44]. Recent publications have indicated that miRNA is present in body fluids and might present a new target for infectious diseases screening [45]. A previous study also observed that BU patients have detectable levels of mycolactone in their body fluids (blood/serum) [46], which might provide a new target for the development of a new diagnostic tool. For a potential mycolactone biosensor, applications using graphene template could be explored. Through computer simulation, the surface of graphene could be functionalised as a substrate for biomolecule scavenging for potential biosensor applications [47]. This study also proposes a mycolactone and graphene interaction study using computational simulation for possible application as a biosensor for BU diagnosis.

Lastly, the widespread use of the Bacillus Calmette–Guérin (BCG) vaccine for TB and probable environmental exposure to other mycobacteria has presented research challenges due to cross reactivity in all the BU studies in Africa [15]. By comparison in Australia, which also reports BU disease, there is the potential to develop a CMI and serology-based assays as the population contains a significant proportion of people naïve to BCG vaccination. Due to public health awareness and subsequent national TB eradication program conducted from the late 1940s, Australia currently has one of the lowest notification rates for TB worldwide and mass BCG vaccination has halted since the mid-1980s [48]. This has created a BCG naïve population in the Australian BU cohort, providing an opportunity for further screening to study cross-reactivity issues.

These suggestions and recommendations have also been considered elsewhere [26].

### Conclusions

Based on this systematic review, pathogen-derived protein preparations appear to perform better at discriminating *M*. *ulcerans* infected patients from non-infected study controls when used in CMI and serology-based assays. We identified a need to standardise study design, protocols for reagent generation and the test design in diagnosis for *M*. *ulcerans* disease. We found a paucity of published articles attempting to develop CMI and serology-based tests for the disease. We recommend further investigations into the development of *M*. *ulcerans* disease specific and sensitive tests.

## Supporting information

S1 PRISMA Checklist (DOC)

S1 Text (DOCX)

S1 Data (XLSX)
