# Supplementary material for: Cell-mediated and serology-based tests for Mycobacterium ulcerans disease: A systematic review and meta-analysis
Source: PLoS Negl Trop Dis. 2020 Apr 6;14(4):e0008172. doi: 10.1371/journal.pntd.0008172 (PMC7162525; doi:10.1371/journal.pntd.0008172)
Supplement: S1 Text — (DOCX) [file pntd.0008172.s002.docx]

**Table A in S1 Text: PubMed**

| **Search** | **Query** | **Items found** |
| --- | --- | --- |
| [#1](http://www.ncbi.nlm.nih.gov/pubmed/advanced) | **Buruli ulcer and diagnosis** | **396** |
| #2 | **Buruli ulcer and serological screening test** | **2** |
| #3 | **Buruli ulcer and cell-mediated assay** | **4** |
| #4 | ***Mycobacterium ulcerans* and diagnosis** | **484** |
| #5 | ***Mycobacterium ulcerans* and serological screening test** | **2** |
| #6 | ***Mycobacterium ulcerans* and cell-mediated assay** | **5** |

**Table B in S1 Text: Web of Science**

| **Search** | **Query** | **Items found** |
| --- | --- | --- |
| [#1](http://www.ncbi.nlm.nih.gov/pubmed/advanced) | **Buruli ulcer and diagnosis** | **212** |
| #2 | **Buruli ulcer and serological screening test** | **1** |
| #3 | **Buruli ulcer and cell-mediated assay** | **8** |
| #4 | ***Mycobacterium ulcerans* and diagnosis** | **229** |
| #5 | ***Mycobacterium ulcerans* and serological screening test** | **1** |
| #6 | ***Mycobacterium ulcerans* and cell-mediated assay** | **3** |
